# Effect of Particle Size and Crystal Surface of CeO_2_ on the Catalytic Combustion of Benzene

**DOI:** 10.3390/ma13245768

**Published:** 2020-12-17

**Authors:** Zhuo Wang, Zhu Chen, Jie Zheng, Shufeng Zuo

**Affiliations:** Zhejiang Key Laboratory of Alternative Technologies for Fine Chemicals Process, Shaoxing University, Shaoxing 312000, China; zhuowang97@163.com (Z.W.); czliving@foxmail.com (Z.C.); zj18258083688@163.com (J.Z.)

**Keywords:** cerium oxide, benzene, catalytic combustion, particle size, crystal surface

## Abstract

In this study, three kinds of CeO_2_ were synthesized, and supported PdO_x_ (x = 0,1) catalysts were prepared for benzene catalytic combustion. The samples were characterized by XRD, N_2_ adsorption/desorption, HRTEM, XPS and H_2_-TPR. The results show that three kinds of CeO_2_ with different structures can be formed by different preparation methods. This is mainly reflected in the differences in pore structure, particle size and crystal plane. CeO_2_-DC obtained from directly calcined Ce(NO_3_)_3_·6H_2_O had the largest pore volume and pore diameter and smallest particle size. CeO_2_-DC was mainly exposed to the (200) plane. Combined with the results of the ability test, it could be concluded that when Pd^2+^ and Pd^0^ exist at the same time, the activity increases with an increase in the proportion of Pd^2+^. Meanwhile, the structure of CeO_2_ affects the formation of oxygen vacancies, thereby affecting the adsorption and degradation of benzene. This article reveals that the particle size, crystal planes, oxygen vacancies and proportion of Pd^2+^ have a great impact on the catalytic combustion of benzene and allow a more comprehensive understanding of the structure–activity relationship, which can guide us to design high-efficiency catalysts targeted to obtain suitable CeO_2_-based catalysts for the catalytic combustion of benzene.

## 1. Introduction

Volatile organic compounds (VOCs) are a main component of air pollution and have been increasing rapidly in recent years. Most VOCs not only harm the environment but also threaten human health due to their toxicity and carcinogenicity [1,2,3]. Aromatic hydrocarbons, as a kind of VOCs, are considered to cause great harm to the environment and are usually toxic and carcinogenic [4,5]. Benzene is a kind of carcinogen, which can be determined to be carcinogenic. Toluene, ethylbenzene and xylene are all possible carcinogens [6]. Among the aromatic hydrocarbons, benzene, toluene, ethylbenzene and xylene (BTEX) constitute the majority of the total industrial emissions [7]. Therefore, it is very urgent to develop an effective and suitable method to reduce the total amount of VOCs. The main methods for dealing with VOCs include adsorption, membrane separation, absorption and oxidative approaches (thermal incineration, biological degradation, photocatalytic decomposition, nonthermal plasma oxidation and catalytic combustion) [8,9,10,11,12,13]. Catalytic combustion has become the most widely used technology because of its characteristics of high catalytic efficiency, low energy consumption and low secondary pollution [14,15,16].

Catalytic combustion is a typical gas–solid catalytic reaction. In the reaction, VOCs need to be adsorbed on the surface of the catalyst, and then a catalytic degradation reaction is carried out. Supports with a high specific surface area (*S*_BET_) and rich pore structure can improve the adsorption property and the catalytic performance of VOCs [8]. Thus, it is important to identify excellent supports for the catalytic combustion of VOCs. Common supports include γ-Al_2_O_3_, cordierite, molecular sieves and pillared clays [17,18,19,20,21]. These supports have large *S*_BET_ and pore volume (*V*_P_) but have no activity or low activity. Due to the low activity of the support, only the proportion, type and loading method of the active component can be improved, which limits the improvement of catalyst performance in the catalytic combustion of VOCs. In recent years, some researchers have found that using active supports can further promote the performance of catalysts [22,23,24,25]. For example, Jablonska et al. synthesized a new HY zeolite that can completely catalyze methanol combustion at 500 °C. After loading Pd, it can completely convert methanol below 150 °C, which is greatly improved compared with Pd/Al_2_O_3_ [25]. Li et al. synthesized a type of CoAl mixed oxide that can completely catalyze toluene conversion at 345 °C. After loading Pt, its activity is greatly improved, and toluene can be completely burned at 230 °C [26]. Therefore, the use of active supports instead of traditional supports provides a development direction for future research.

Compared with other active supports, rare-earth elements have many advantages in catalytic materials, such as increasing oxygen storage and releasing capacity, adjusting catalytic active sites and improving the dispersion of active components and the thermal stability of catalysts. Therefore, rare-earth oxides, as active supports, have been widely used in VOCs catalytic combustion reactions [24,27]. Wu et al. synthesized a series of LaCo composite oxides for the catalytic combustion of toluene. By introducing La, the valence state of Co can be adjusted, and the formation of oxygen vacancies can be promoted, which causes the catalyst to have good catalytic performance for toluene combustion [28]. Wu et al. prepared LaFeO_3_ perovskite oxide for the catalytic combustion of toluene, which showed high catalytic activity [29]. Cerium oxide (CeO_2_), one of the most significant rare-earth oxides, has attracted much attention in recent decades. Many researchers have applied CeO_2_ as a catalyst in the catalytic combustion of VOCs [27,30,31,32]. Chen et al. synthesized a Ce metal–organic frameworks (MOF) catalyst which showed good catalytic activity for toluene oxidation [33]. Wang et al. synthesized a honeycomb CeO_2_ catalyst. It had great differences in morphology and reducibility and exhibited high activity in the oxidation of o-xylene [24]. However, CeO_2_ catalysts cannot achieve ideal catalytic performance due to the limitation of structure and physicochemical properties. Therefore, it is still necessary to use CeO_2_ as a support to prepare catalysts. For combustion of light hydrocarbons, several works have pointed out that noble metals as active components exhibited excellent performance of catalytic combustion. Guo et al. successfully prepared a 3D ordered mesoporous CeO_2_ catalyst loaded with biogenic Pd nanoparticles for the catalytic combustion of benzene. The experimental results show that the catalyst has good catalytic activity and can achieve 90% catalytic conversion at 187 °C [34]. Abbasi et al. prepared nanostructured Pt/Al_2_O_3_–CeO_2_ catalysts for catalytic combustion of VOCs, and they exhibited high catalytic activity [35]. Lei et al. synthesized a Pd/CeO_2_ catalyst for methane catalytic combustion. The experimental results show that the catalytic activity of the catalyst is greatly improved after loading Pd, which can completely catalyze combustion at 360 °C [31]. Nowadays, many researchers focus on the selection of active components and the role of rare-earth oxides as promoters. However, there is little research on the effects of pore structure, particle size, crystal surface and other physical indicators on the performance of catalysts and the structure–activity relationship.

In this paper, three kinds of CeO_2_ with different physicochemical properties were prepared and used to prepare Pd/CeO_2_ catalysts. Then, the influence of these properties on the catalytic combustion of benzene was discussed. XRD, N_2_ adsorption/desorption, HRTEM, XPS and H_2_-TPR tests were used to systematically analyze the structure–activity relationship.

## 2. Materials and Methods

### 2.1. Support Preparation

CeO_2_ (particle size: 20–50 nm, 99.5% purity) was purchased from Aladdin and used as received, which is denoted CeO_2_-P. Ce(NO_3_)_3_·6H_2_O (99.5% purity, Aladdin) was directly calcined at 500 °C for 2 h to obtain CeO_2_, which is called CeO_2_-DC. Another CeO_2_ was made by thermal decomposition of Ce metal‑organic frameworks (MOF), which is named CeO_2_-MOF. The preparation method of Ce-MOF has been described in our previous articles [36].

### 2.2. Catalyst Preparation

The catalysts loaded with Pd nanoparticles (0.2 wt.%) were synthesized by high-temperature liquid reduction [37]. First, 2.0 g of CeO_2_ and 100 mL of ethylene glycol (AR, 98%, Aladdin, Xi’an, China) were added to a three-necked flask with stirring for 30 min, and then 0.4 mL of H_2_PdCl_4_ (10 mg/mL) was added into the three-necked flask and stirred for 12 h. Then, the pH was controlled at 11.0. The reaction was heated to 165 °C for 3 h, and then the temperature dropped to room temperature. The product was washed with distilled water twice, then washed with ethanol (AR, 99.7%, Aladdin, Xi’an, China) once and then dried for 12 h at 60 °C. After that, the product was calcined in a muffle furnace (Shanghai Pudong Yuexinxue Instrument Factory, Shanghai, China) at 500 °C for 2 h to obtain a 0.2% Pd/CeO_2_ catalyst. Pd/CeO_2_-P, Pd/CeO_2_-DC and Pd/CeO_2_-MOF were obtained by the above method using CeO_2_-P, CeO_2_-DC and CeO_2_-MOF, respectively.

### 2.3. Catalytic Performance Evaluation

The activity of the catalysts was evaluated on a WFS-3010 microreactor (Xianquan, Tianjin, China) with a benzene concentration of 1000 ppm and a space velocity of 20,000 h^−1^. An amount of 200 mg catalyst (40–60 mesh) was loaded in the quartz reactor and pretreated at 400 °C in air for 0.5 h. After that, 1000 ppm benzene was added for the activity test. Online gas chromatography (Shimadzu, GC-14C, Kyoto, Japan) with a hydrogen flame ionization detector (FID) was used to detect the concentration of the organic substance entering the chromatograph. The chromatographic working conditions were as follows. The temperatures of the column and vaporization chamber were 80 and 120 °C, respectively. An N2000 online chromatography workstation (Saizhi Technology Co., Ltd, Hangzhou, China) was used to record data. The durability of catalysts was tested under the same working conditions of the activity evaluation, and the catalytic time was as long as 100 h.

### 2.4. Catalyst Characterization

The structure of catalysts and supports was characterized using XRD (PANalytical, Almelo, The Netherlands, Cu Kα radiation, 40 kV, 40 mA, speed of 0.02°/s). The particle size of samples was calculated from the XRD data using the Scherrer formula (Equation (1)):

(1) $$D=\frac{K\Upsilon }{B\cos\theta }$$

where:

D = Average thickness of particle perpendicular to crystal plane (nm);
K = Scherrer constant;
γ = X-ray wavelength (0.154 nm);
B = Full width at half maxima (FWHM);
θ = Diffraction angle.

A Tristar II 3020 apparatus (Micromeritics Company, Norcross, GA, USA) was used to determine the nitrogen adsorption and desorption isotherms at −195.8 °C. The Brunauer-Emmett-Teller (BET) model was used to measure the *S*_BET_. Meanwhile, the Barrett-Joyner-Halenda (BJH) method was used to calculate the pore size and average pore diameter. Before the adsorption process, the samples were kept under vacuum at 200 °C for 4 h.

The morphology of the supports and the catalysts was obtained using high-resolution transmission electron microscopy (HRTEM, JEOL-2010F, Tokyo, Japan) operated at 200 kV. The chemical composition of the Pd/CeO_2_ catalyst was detected using energy-dispersive X-ray spectroscopy (EDS) with an Oxford INCA instrument (OXFORD instruments, Abingdon, UK).

The valence states and the elemental proportions of samples were analyzed by X-ray photoelectron spectroscopy (XPS) (Thermo Fisher Scientific, Waltham, MA, USA) on a Thermo ESCALAB 250 with Al Kα (hν = 1486.8 eV) as the excitation source. C1s was used as the internal reference to calibrate electron energies.

Temperature-programmed reduction (H_2_-TPR) experiments were carried out in a CHEMBET-3000 apparatus prior to the measurement, and 200 mg catalyst was put into the quartz reactor and pretreated in air at 300 °C for 0.5 h. After being cooled to room temperature, the catalyst was raised from room temperature to 900 °C under H_2_ flow (5 vol.% in Ar, 40 mL/min) at a heating rate of 15 °C/min. A gas chromatograph (TCD) (Shimadzu, GC-14C, Kyoto, Japan) was used to record and analyze data.

## 3. Results and Discussion

### 3.1. Catalytic Activity Evaluation

**Figure 1:**
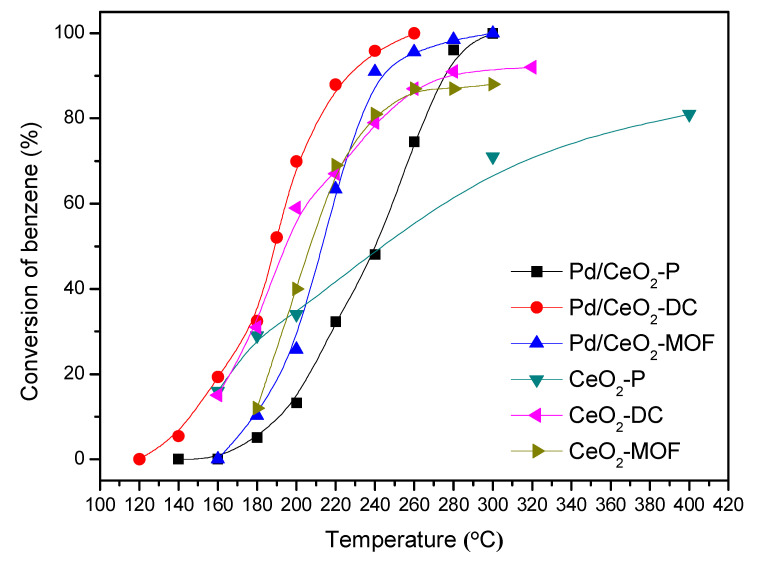
Effect of different CeO_2_ and Pd/CeO_2_ on catalytic combustion of benzene.

The catalytic activity of the CeO_2_ supports and Pd/CeO_2_ catalysts is shown in Figure 1. The results show that the performance of three different CeO_2_ samples was obviously different, which indicated that the ability of CeO_2_ was closely related to the preparation method. The catalytic combustion activity of benzene followed the sequence CeO_2_-DC > CeO_2_-MOF > CeO_2_-P. The conversion rate of CeO_2_-DC for benzene reached 91.7% at 320 °C. After loading Pd, the catalytic combustion ability of benzene was significantly improved. All the catalysts showed better catalytic ability than single CeO_2_, indicating that the active component of PdO_x_ plays an important role in benzene catalytic combustion. Catalytic activity decreases in the following order: Pd/CeO_2_-DC > Pd/CeO_2_-MOF > Pd/CeO_2_-P. The complete conversion temperatures of benzene using Pd/CeO_2_-MOF and Pd/CeO_2_-P were 280 and 300 °C, respectively. Pd/CeO_2_-DC showed the best performance of catalytic combustion, which could completely convert benzene at 260 °C. The catalytic activity data of the samples and related catalysts for benzene catalytic combustion are compared in Table S1. Although the Pd/CeO_2_ catalyst synthesized by Guo et al. can achieve 90% benzene catalytic combustion at 187 °C, they used a higher content of Pd (2 wt.%) than Pd/CeO_2_-DC [34]. Therefore, considering the comprehensive cost, Pd/CeO_2_-DC has high application value.

### 3.2. Durability Test

**Figure 2:**
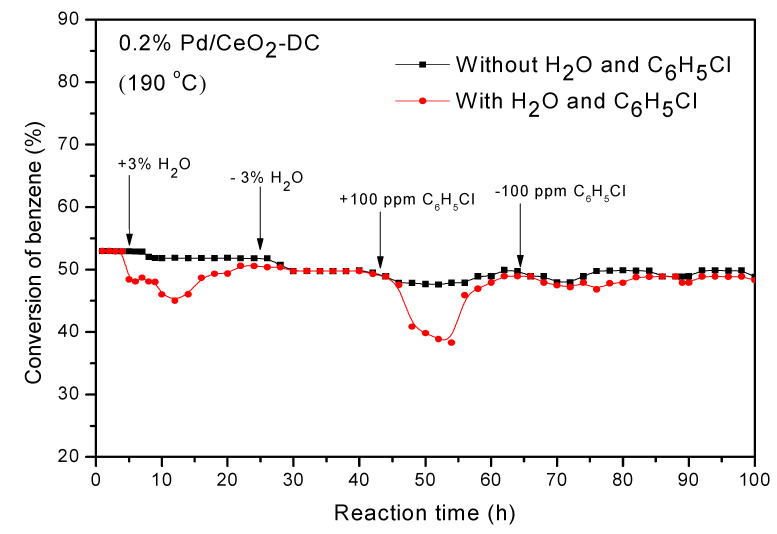
Durability test for catalytic combustion of benzene over the 0.2% Pd/CeO_2_-DC catalyst.

The temperature of the conversion rate at 52% was selected for the life test of the catalyst. Figure 2 shows the results of the durability test of Pd/CeO_2_-DC at 190 °C. After 100 h of reaction, the activity of the catalyst remained at approximately 49%, with no obvious decrease. It exhibited exceptional stable catalytic activity. Industrial chlorine-containing VOCs and water were widely distributed in waste gases. Figure 2 shows that when 3 vol.% water was added, its catalytic activity decreased because of competitive adsorption [38]. After introducing chlorobenzene at 100 ppm into the system, the catalytic activity further decreased. However, after water vapor and chlorobenzene were removed, the activity of the catalyst returned to its original level. The results show that the Pd/CeO_2_-DC catalyst had a certain ability to resist chlorine poisoning that may be due to the interaction between PdO_x_ and CeO_2_-DC, which can timely release the combustion products of chlorobenzene and prevent chlorine poisoning. Meanwhile, it exhibited good resistance to moisture conditions. In addition, the catalyst maintained its catalytic activity over a long reaction time of 100 h.

### 3.3. XRD Analysis

**Figure 3:**
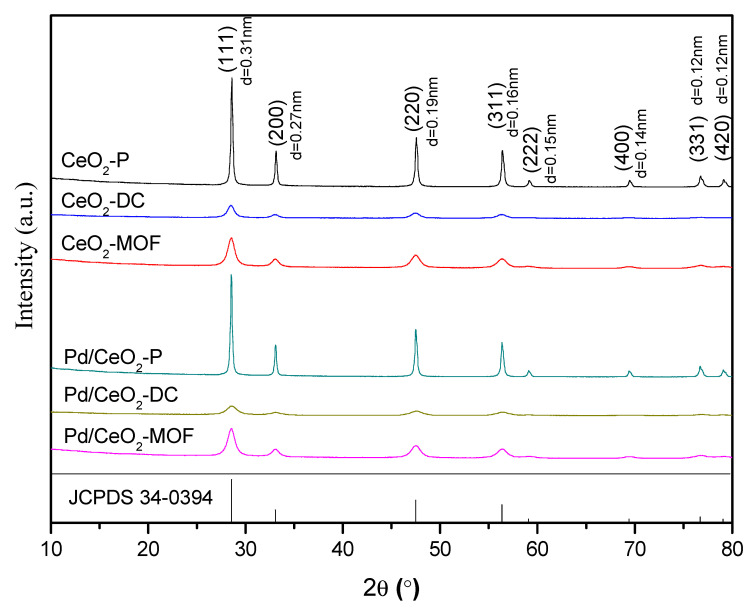
XRD patterns of the samples.

Figure 3 (XRD patterns of supports and catalysts) shows that the patterns of samples have characteristic diffraction peaks of CeO_2_. All samples exhibited several peaks at 28.7°, 33.2°, 47.7°, 56.6°, 59.4°, 69.7°, 77.1° and 79.5°, pointing to the (111), (200), (220), (311), (222), (400), (331) and (420) crystal planes, respectively (JCPDS No. 34-0394) [26]. The XRD spectra of the precursor Ce-MOF are shown in Figure S1. From Figure 3, it can be found that the intensity and FWHM of the diffraction peak are different and decrease in the following order: CeO_2_-P > CeO_2_-MOF > CeO_2_-DC. According to the Scherrer formula, the average particle size of the samples can be calculated as follows: CeO_2_-P (34 nm) > CeO_2_-MOF (11 nm) > CeO_2_-DC (8 nm). This result indicates that CeO_2_-DC prepared by direct calcining had the smallest particle size. As shown in Figure 3, it can be concluded that the crystallinity of CeO_2_-P is the highest and that of CeO_2_-DC is the lowest. Combined with their particle size, it can be inferred that the smaller the particle size of CeO_2_, the poorer the crystallinity. It is well known that the lattice defects of CeO_2_ increase with decreasing particle size, so the smaller particle size of CeO_2_ will lead to the formation of surface lattice defects and the generation of reactive oxygen species [33]. However, no diffraction peak for the PdO_x_ phase could be found, which may be attributed to the low loading content and the high dispersion of Pd.

### 3.4. HRTEM Analysis

**Figure 4:**
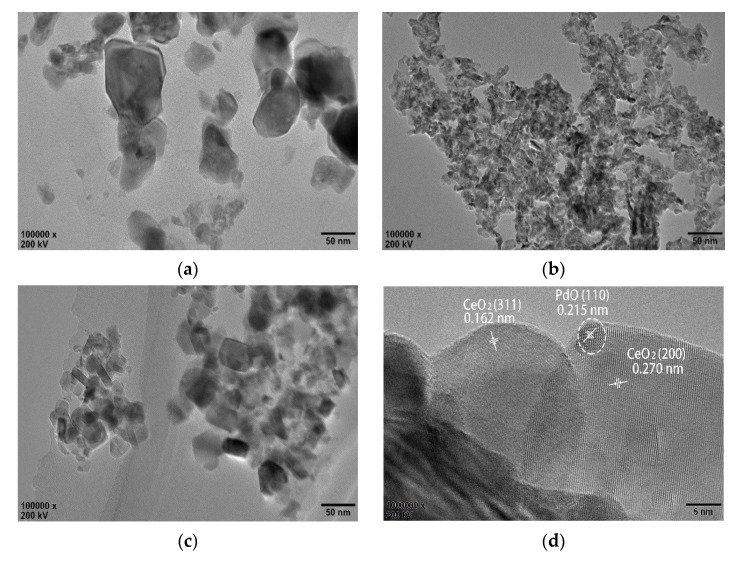

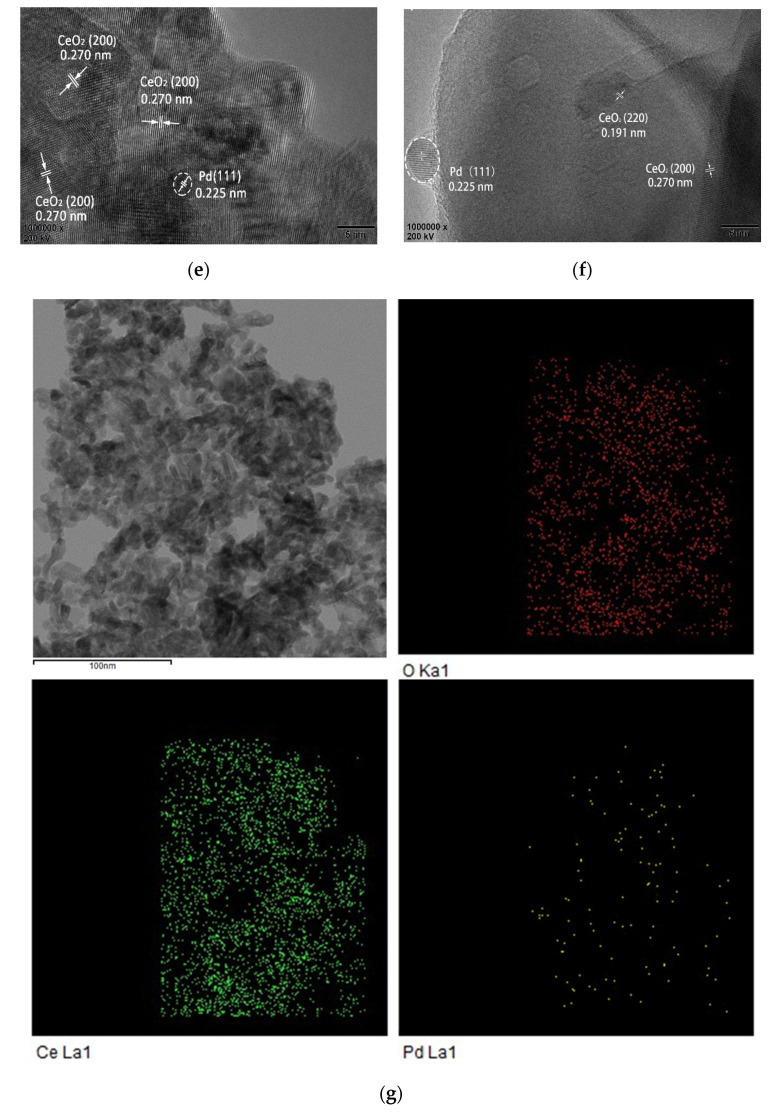
HRTEM images: (**a**) CeO_2_-P; (**b**) CeO_2_-DC; (**c**) CeO_2_-MOF; (**d**) Pd/CeO_2_-P; (**e**) Pd/CeO_2_-DC; (**f**) Pd/CeO_2_-MOF; (**g**) EDS mapping images of Pd/CeO_2_-DC (The red, green and yellow dots represent O, Ce and Pd elements, respectively).

Figure 4 shows the morphology and crystal plane structure of CeO_2_ supports and their Pd-based catalysts. Figure 4a–c show that all CeO_2_ synthesized by different preparation methods have the morphology of nanoparticles and show an irregular shape. The particle sizes of CeO_2_-P, CeO_2_-DC and CeO_2_-MOF were 30–60, 3–10 and 10–30 nm, respectively. It was found that the particle size of CeO_2_-DC was significantly smaller than that of CeO_2_-P and CeO_2_-MOF, which was consistent with the results of XRD analysis. The fine structure of three different Pd/CeO_2_ was observed when the resolution was further improved. We can observe the lattice fringes of PdO_x_ and CeO_2_, as shown in Figure 4d–f. The lattice fringe spacing was calculated by Image J digital micrograph software. For Pd/CeO_2_-P, the lattice fringe spacing is 0.215 nm, corresponding to the PdO (110) crystal plane (JCPDS No. 41-1107), and CeO_2_-P mainly exposes the (311) and (200) crystal planes. For Pd/CeO_2_-DC and Pd/CeO_2_-MOF, the lattice fringe spacing is 0.225 nm, corresponding to the Pd (111) crystal plane (JCPDS No. 46-1043); CeO_2_-DC mainly exposes (200) crystal planes with a lattice fringe spacing of 0.270 nm; and CeO_2_-MOF has two crystal plane spacings of 0.191 and 0.270 nm, corresponding to the (220) and (200) crystal planes. The exposed crystal surface may affect the formation of oxygen vacancies, and different Pd/CeO_2_ should have different chemical properties [33]. Through catalytic activity testing, we can assume that the (200) crystal plane of CeO_2_ may play an important role in benzene catalytic combustion. The morphology of the recovered catalyst is shown in Figure S2. It can be found that the morphology of the catalyst has no obvious change after use, indicating that the catalyst has good stability.

In addition, the EDS spectrum of Pd/CeO_2_-DC had Pd, O and Ce signals, as shown in Figure 4g, indicating that the active components of PdO_x_ have been successfully loaded and highly dispersed in CeO_2_-DC and the particle size of PdO_x_ nanoparticles is about 2–3 nm.

### 3.5. N_2_ Adsorption/Desorption Analysis

**Figure 5:**
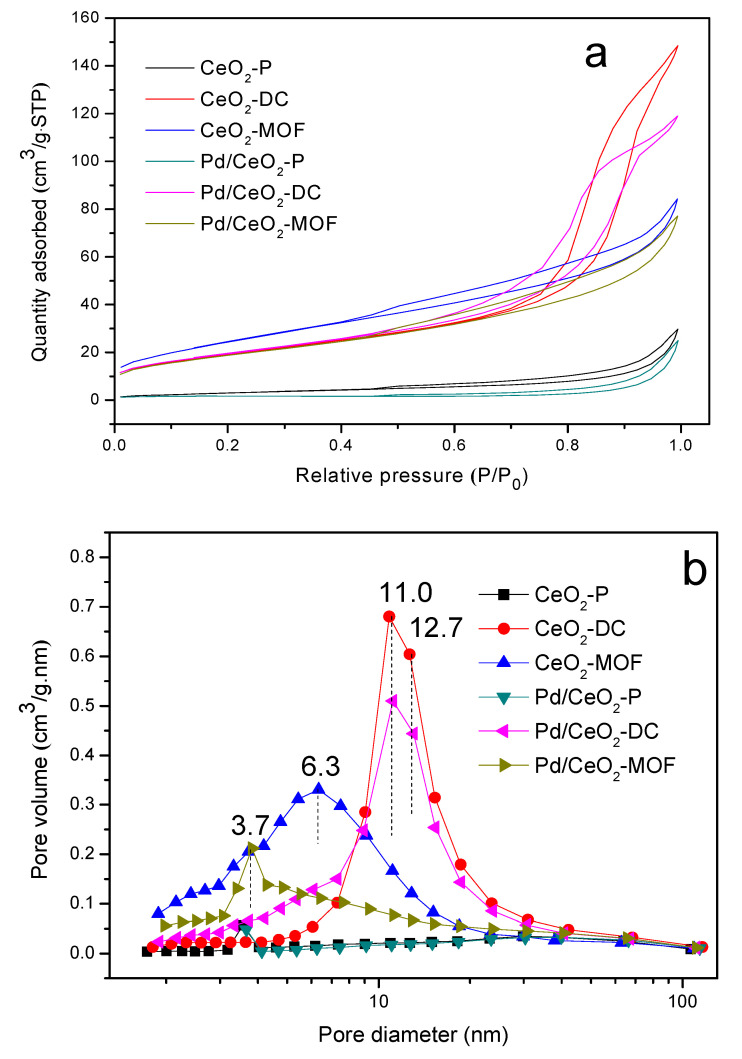
Textural properties of the samples: (**a**) N_2_ adsorption/desorption isotherms; (**b**) pore size distributions.

The pore size distribution and *S*_BET_ of the catalyst have a great influence on the activity of catalytic combustion. It can be measured by N_2_ adsorption/desorption, and the results are shown in Figure 5. It can be clearly seen in Figure 5a that all the samples showed a type IV isotherm, which indicates that there are mesoporous structures in the materials [39,40]. CeO_2_-P and CeO_2_-MOF showed a H3 hysteresis loop appearing at P/P_0_ = 0.4–1.0, indicating that there are slit-shaped pores in the sample [41]. Additionally, CeO_2_-DC exhibited a H1 hysteresis loop, indicating that there is a cylindrical order in the sample. The pore size distribution curve is shown in Figure 5b. From the pore size distribution curves, it can be clearly found that the average pore size of CeO_2_-DC is approximately 11.0–12.7 nm and the *V*_P_ is 0.23 cm^3^/g. Meanwhile, the pore size does not change after loading PdO_x_ nanoparticles, but the *V*_P_ decreases slightly. Table 1 summarizes the physical properties of samples according to the N_2_ adsorption/desorption isotherms. From Table 1, we can conclude that the *S*_BET_ of three different CeO_2_ is arranged as follows: CeO_2_-MOF > CeO_2_-DC > CeO_2_-P. After the introduction of PdO_x_ nanoparticles, *V*_P_ decreased in varying degrees, and some catalysts formed a microporous structure, which may be due to the blockage of the pore channels by PdO_x_ nanoparticles. However, the *S*_BET_ of CeO_2_-DC increases after loading with PdO_x_ nanoparticles, which may be due to the fact that PdO_x_ nanoparticles are mainly distributed on the surface of CeO_2_-DC. The results of EDS in Figure 4g also show that PdO_x_ is mainly distributed on the surface of CeO_2_-DC and highly dispersed. Pd/CeO_2_-DC has the highest *S*_BET_ (80.4 m^2^/g) and the largest *V*_P_ (0.21 cm^3^/g) among all catalysts. It is well known that higher *S*_BET_ and *V*_P_ can provide more active sites and promote catalytic activity. The results are consistent with the experimental results of the catalytic combustion performance.

### 3.6. XPS Analysis

**Figure 6:**
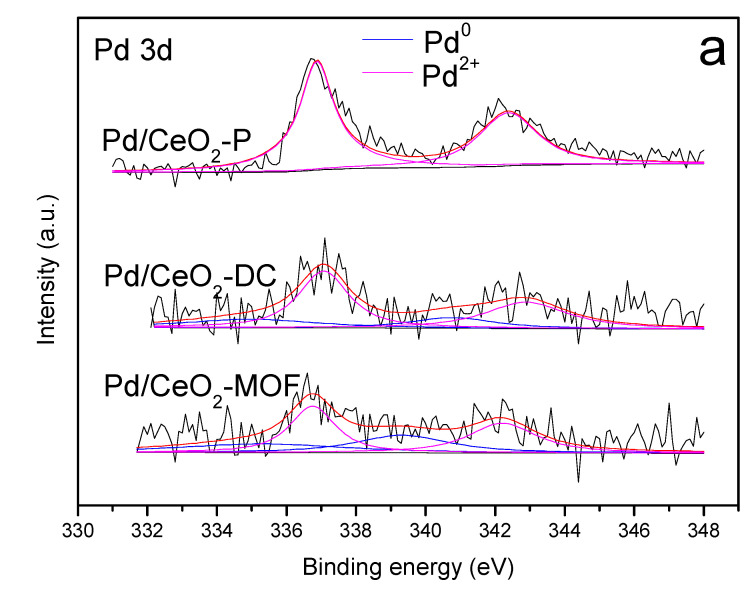

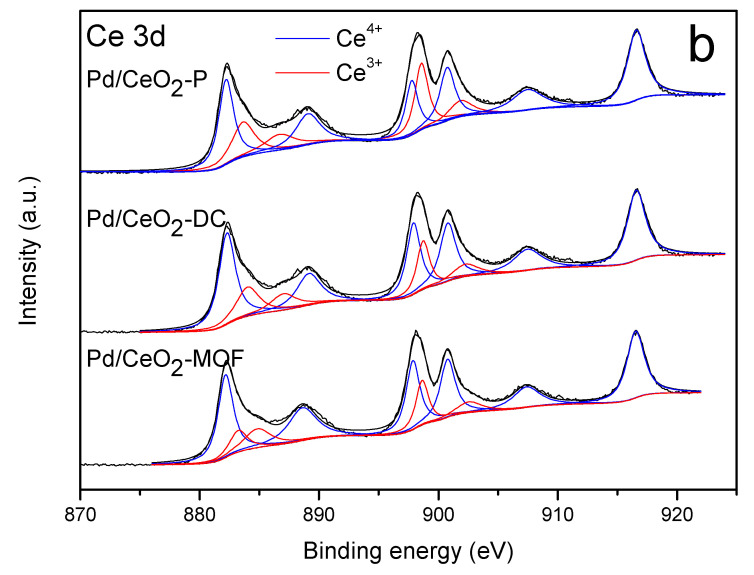
XPS spectrum: (**a**) Pd 3d; (**b**) Ce 3d.

The valence states of the elements on the surfaces of materials can be investigated using the XPS technique. Figure 6a shows the Pd 3d spectra of the catalysts. It was reported that the complex spectrum of Pd 3d can be decomposed into four peaks associated with the two spin orbitals. The peaks of 339.3–342.9 and 335.3–337.1 eV can be allocated to Pd 3d_3/2_ and Pd 3d_5/2_, respectively [30]. It is obvious that Pd^0^ and Pd^2+^ were detected in the prepared Pd/CeO_2_-DC and Pd/CeO_2_-MOF catalysts, but only Pd^2+^ was detected in the prepared Pd/CeO_2_-P catalyst. The percentage of Pd^2+^ species in the catalyst was obtained by calculating the fitted area of Pd^2+^/(Pd^2+^ + Pd^0^). The concentration of Pd^2+^ in catalysts is reduced in the following order: Pd/CeO_2_-P (100%) > Pd/CeO_2_-DC (71.1%) > Pd/CeO_2_-MOF (57.1%). Many researchers found that Pd^2+^ plays an important role in hydrocarbon oxidation, indicating that Pd^2+^ is more active than Pd^0^ in the reaction [42]. However, it is worth noting that compared with other catalysts, Pd/CeO_2_-P exhibited relatively low activity, indicating that single Pd^2+^ has a negative effect on benzene catalytic combustion [43].

Figure 6b shows the Ce 3d spectra of the catalysts. Ce mainly exists in the form of Ce^3+^ and Ce^4+^ [44]. According to the equation of Ce^3+^/(Ce^3+^ + Ce^4+^), the ratio of Ce^3+^ on the catalyst was calculated based on the peak area. To compare the concentration of Ce^3+^ on the supports and catalysts, the concentration of Ce^3+^ on the CeO_2_ support was calculated. The results show that the proportion of Ce^3+^/(Ce^3+^ + Ce^4+^) on supports is reduced in the following order: CeO_2_-DC (22.5%) > CeO_2_-MOF (21.9%) > CeO_2_-P (17.8%). After loading PdO_x_, the concentration of Ce^3+^ on the CeO_2_ surface increased, which indicates that there is a strong interaction between PdO_x_ and CeO_2_. Pd/CeO_2_-DC had the highest proportion of Ce^3+^/(Ce^3+^ + Ce^4+^). The proportion of Ce^3+^/(Ce^3+^ + Ce^4+^) decreased in the order: Pd/CeO_2_-DC (24.8%) > Pd/CeO_2_-MOF (23.1%) > Pd/CeO_2_-P (18.1%). This was consistent with the higher activity of Pd/CeO_2_-DC. The corresponding results are listed in Table 2. It has been reported that Ce^3+^ can promote the formation of oxygen vacancies [35]. Therefore, a higher ratio of Ce^3+^ means more oxygen vacancies on the surface of the catalyst, which can promote the interaction between PdO_x_ and CeO_2_, which is crucial for redox performance and catalytic activity. It is well known that CeO_2_ has good oxygen storage performance and oxygen in the CeO_2_ phase can be moved. Due to the strong interaction between PdO_x_ and CeO_2_, Pd/CeO_2_-DC can transfer oxygen from CeO_2_ to PdO_x_, resulting in the appearance of more Ce^3+^ and Pd^2+^. Combined with the data of activity evaluation, it can be found that the difference in activity of Pd/CeO_2_ catalysts with the same preparation method and the same Pd loading amount is mainly due to the different structure of CeO_2_. CeO_2_ prepared by different methods has different particle sizes and exposed crystal planes, which leads to different binding abilities between PdO_x_ and CeO_2_, resulting in the change of the valence state of Pd and CeO_2_ on the catalyst surface, thus affecting the catalytic activity of the catalysts.

### 3.7. H_2_-TPR Analysis

**Figure 7:**
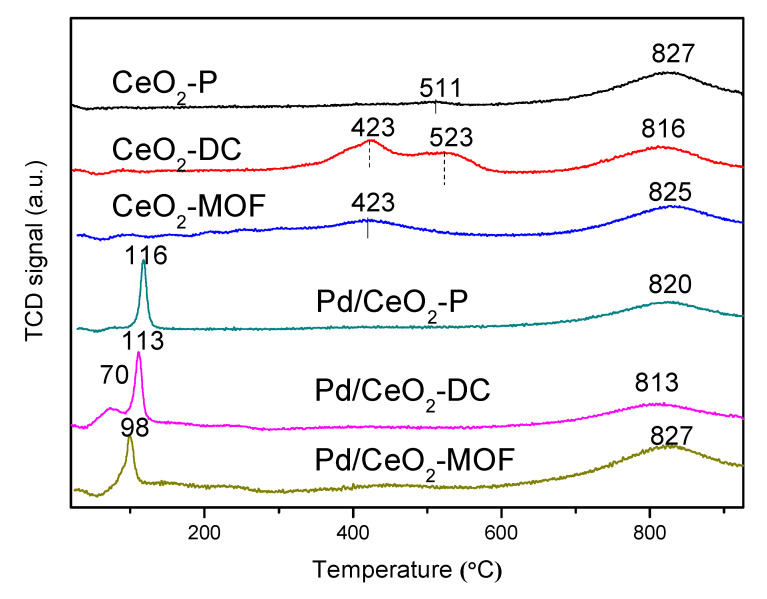
H_2_-TPR curves of the samples.

To study the redox ability of different CeO_2_ and their Pd/CeO_2_ catalysts, H_2_-TPR was used. As shown in Figure 7, CeO_2_ exhibits one or two reduction peaks at temperatures below 600 °C, which are attributed to the reduction of CeO_2_ surface oxygen and subsurface oxygen. When the temperature is higher than 800 °C, the CeO_2_ lattice oxygen is reduced. The total hydrogen consumption follows the sequence CeO_2_-DC > CeO_2_-MOF > CeO_2_-P. The results show that CeO_2_-DC has the best redox capacity, which is consistent with the catalytic activity. After loading PdO_x_, a new peak appeared near 100 °C, and the peak disappeared between 423 and 523 °C, which indicates that there is a strong interaction between PdO_x_ and CeO_2_ which promotes the reduction of CeO_2_. The peak at low temperature is caused by the reduction of PdO_x_ and the co-reduction of oxygen adsorbed on the CeO_2_ surface. Obviously, although Pd/CeO_2_-DC shows a small acromion below 100 °C, neither Pd/CeO_2_-P nor Pd/CeO_2_-MOF has an acromion, which can be attributed to the strong binding with Pd-O-Ce, resulting in a higher PdO_x_ reduction temperature. Therefore, the first reduction temperature of Pd/CeO_2_-DC is the lowest (70 °C); that is, the PdO_x_ species on CeO_2_-DC is relatively easy to reduce, which leads to maintaining the palladium metal state and catalytic oxidation of benzene activity. It has been reported that the reducibility of catalysts is closely related to their catalytic activity, and catalysts with lower reduction temperatures usually show higher catalytic activity.

## 4. Conclusions

In this study, three kinds of CeO_2_ were obtained by purchase, calcining of Ce(NO_3_)_3_·6H_2_O and thermal decomposition of Ce-MOF, which exhibited different pore structures, particle sizes and crystal planes. CeO_2_-DC had the largest pore volume and the smallest particle size, which promotes the formation of oxygen vacancies. Meanwhile, the Pd/CeO_2_ catalysts were synthesized by high-temperature liquid reduction, and Pd/CeO_2_-DC showed the best catalytic performance, excellent durability and resistance to poisoning in the catalytic combustion of benzene due to the good properties of CeO_2_-DC, which could completely combust benzene at 260 °C. Moreover, the research indicates that the difference in binding ability between the exposed surfaces of CeO_2_ and Pd can affect catalytic activity and that CeO_2_ with the (200) crystal plane may play an important role in the catalytic combustion of benzene. Furthermore, the increase in the Pd^2+^ proportion can promote the catalytic activity when Pd^0^ and Pd^2+^ exist at the same time. These characteristics indicate that the catalyst has great potential in industrial applications. However, we must realize that although this catalyst has shown high catalytic activity in the laboratory, the industrial environment is more complex and changeable. Therefore, further research in environments with high concentrations and multiple mixed VOCs is urgently required.

## Figures and Tables

**Table 1:** Characteristic data of the samples.

Samples	*S*_BET_^a^ (m^2^/g)	*A*_mes_^b^ (m^2^/g)	*V*_P_^c^ (cm^3^/g)	*V*_mic_^d^ (cm^3^/g)
CeO_2_-P	10.7	10.7	0.044	-
CeO_2_-DC	66.3	66.3	0.23	-
CeO_2_-MOF	90.5	90.5	0.14	-
Pd/CeO_2_-P	7.7	5.9	0.039	0.00074
Pd/CeO_2_-DC	80.4	77.3	0.21	0.00059
Pd/CeO_2_-MOF	73.8	73.8	0.13	-

^a^ The BET specific surface area. ^b^ Calculated by the BJH method. ^c^ Total pore volume estimated at P/P_0_ = 0.99. ^d^ Micropore volume calculated by the t-plot method.

**Table 2:** Summary table of XPS characterization result of catalysts.

Catalyst		Pd 3d (eV)		Ce 3d (eV)		Pd^2+^/(Pd^2+^ + Pd^0^) (%)	Ce^3+^/(Ce^3+^ + Ce^4+^) (%)
		Pd^0^	Pd^2+^	Ce^3+^	Ce^4+^		
Pd/CeO_2_-P	3d^3/2^	-	342.4	883.7, 887.3	882.2, 889.3, 897.8	100	18.1
	3d^5/2^	-	336.9	898.6, 901.9	900.7, 907.5, 916.6		
Pd/CeO_2_-DC	3d^3/2^	340.8	342.9	884.0, 887.0	882.3, 889.2, 897.9	71.1	24.8
	3d^5/2^	335	337.1	898.7, 902.2	900.8, 907.4, 916.5		
Pd/CeO_2_-MOF	3d^3/2^	339.3	342.2	883.3, 884.8	882.3, 888.7, 897.9	57.1	23.1
	3d^5/2^	335.3	336.8	898.7, 902.4	900.8, 907.4, 916.5		

## Supplementary Materials

The following are available online at https://www.mdpi.com/1996-1944/13/24/5768/s1, Figure S1: The XRD diffraction pattern of the precursor Ce-MOF-120 of CeO2-MOF, Figure S2: The morphology of recovered Pd/CeO2-DC catalyst, Table S1: The catalytic activity data of the samples and related catalysts for benzene catalytic combustion.
