# Supplementary material for: Effect of Particle Size and Crystal Surface of CeO2 on the Catalytic Combustion of Benzene
Source: Materials (Basel). 2020 Dec 17;13(24):5768. doi: 10.3390/ma13245768 (PMC7766107; doi:10.3390/ma13245768)
Supplement: Supplementary file 1 [file materials-13-05768-s001.pdf]

materials

Article

Supplementary Materials: Effect of Particle Size and Crystal Surface of CeO_2 on the Catalytic Combustion of Benzene

Zhuo Wang, Zhu Chen, Jie Zheng and Shufeng Zuo *

Zhejiang Key Laboratory of Alternative Technologies for Fine Chemicals Process, Shaoxing University, Shaoxing 312000, China; zhuowang97@163.com (Z.W.); czliving@foxmail.com (Z.C.); zj18258083688@163.com (J.Z.)

* Correspondence: sfzuo@usx.edu.cn; Tel.: +86-575-8834-1616

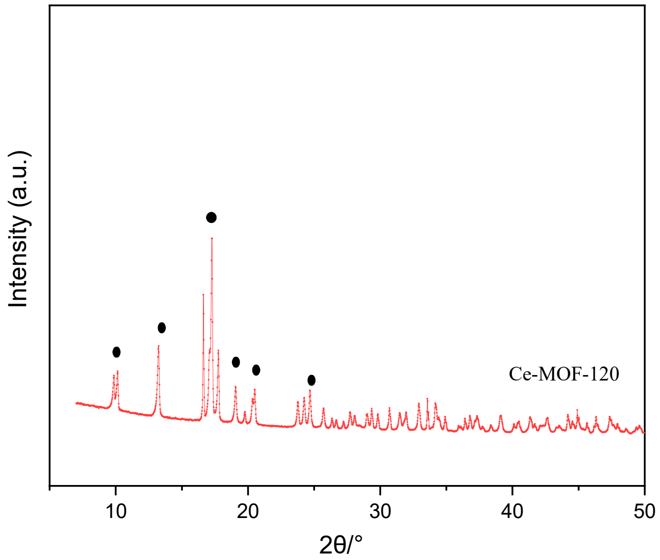

Figure S1. The XRD diffraction pattern of the precursor Ce-MOF-120 of CeO_2-MOF

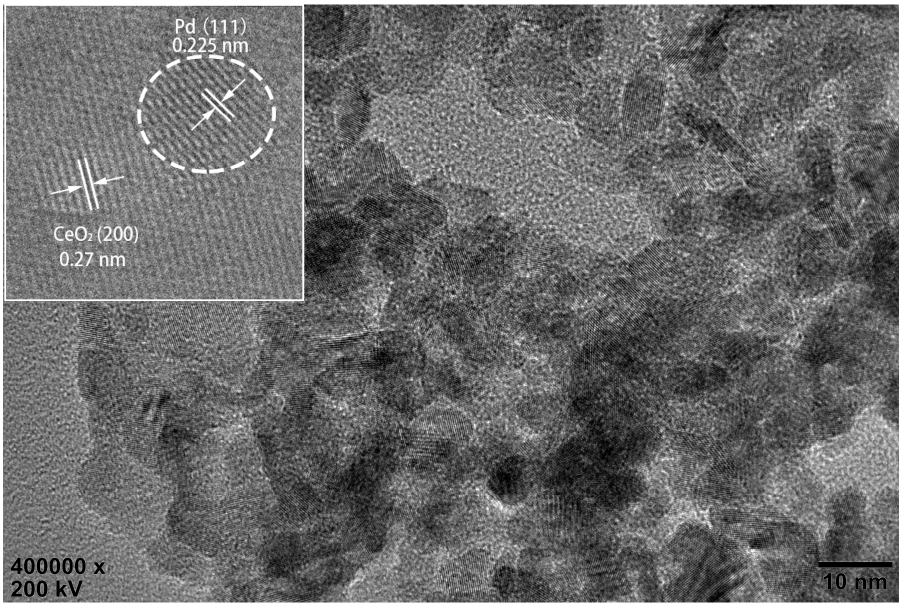

Figure S2. The morphology of recovered Pd/CeO_2-DC catalyst

Table S1. The catalytic activity data of the samples and related catalysts for benzene catalytic combustion.

samples	Reaction conditions			Conversion temperature
	Active sites (wt. %)	VOCs	Concentration (ppm)	
CeO_2-P	-	Benzene	1000	400 (T_{80})
CeO_2-MOF	-	Benzene	1000	320 (T_{90})
CeO_2-DC	-	Benzene	1000	300 (T_{90})
Pd/CeO_2-P	0.2 wt. % Pd	Benzene	1000	300 (T_{100})
CeO_2-MOF	0.2 wt. % Pd	Benzene	1000	280 (T_{100})
Pd/CeO_2-DC	0.2 wt. % Pd	Benzene	1000	260 (T_{100})
Pd/CeO_2 [34]	2 wt. % Pd	Benzene	1000	187 (T_{90})
Pd/Al2O_3-CeO_2 [35]	1 wt. % Pd	Benzene	-	300 (T_{85})

PDF#41-1107: QM=Uncommon(?); d=Diffractometer; I=(Unknown)
Palladinite
PdO

Radiation=CuKa1 Lambda=1.5406 Filter=
Calibration= 2T=29.316-138.984I/Ic(RIR)=
Ref: Level-1 PDF

Tetragonal, P42/mmc(131) Z=2 mp=
CELL: 3.0456 x 3.0456 x 5.3387 <90.0 x 90.0 x 90.0> P.S=
Density(c)=8.209 Density(m)= Mwt= Vol=49.5
Ref: Ibid.

Strong Lines: 2.65/X 2.67/2 1.68/2 1.54/2 1.32/2 2.15/1 1.32/1 1.52/1

2-Theta	d(?)	I(f)	(h k l)	Theta	1/(2d)	2pi/d	n^2
29.316	3.0440	3.0	(1 0 0)	14.658	0.1643	2.0641	
33.549	2.6690	22.0	(0 0 2)	16.774	0.1873	2.3541	
33.836	2.6470	100.0	(1 0 1)	16.918	0.1889	2.3737	
41.927	2.1530	14.0	(1 1 0)	20.963	0.2322	2.9183	
45.138	2.0070	1.0	(1 0 2)	22.569	0.2491	3.1306	
54.715	1.6762	20.0	(1 1 2)	27.357	0.2983	3.7485	
60.218	1.5355	15.0	(1 0 3)	30.109	0.3256	4.0919	
60.782	1.5226	11.0	(2 0 0)	30.391	0.3284	4.1266	
70.521	1.3343	6.0	(0 0 4)	35.260	0.3747	4.7090	
71.251	1.3224	15.0	(2 0 2)	35.626	0.3781	4.7514	
71.457	1.3191	13.0	(2 1 1)	35.728	0.3790	4.7632	
85.534	1.1344	3.0	(1 1 4)	42.767	0.4408	5.5388	
90.834	1.0815	5.0	(2 1 3)	45.417	0.4623	5.8097	
91.353	1.0767	5.0	(2 2 0)	45.677	0.4644	5.8356	
98.721	1.0151	2.0	(3 0 0)	49.360	0.4926	6.1897	
99.720	1.0076	5.0	(1 0 5)	49.860	0.4962	6.2358	
100.263	1.0036	5.0	(2 0 4)	50.131	0.4982	6.2606	
100.938	0.9987	5.0	(2 2 2)	50.469	0.5007	6.2914	
101.161	0.9971	5.0	(3 0 1)	50.581	0.5015	6.3015	
106.236	0.9630	2.0	(3 1 0)	53.118	0.5192	6.5246	
107.805	0.9533	1.0	(2 1 4)	53.902	0.5245	6.5910	
116.528	0.9057	4.0	(3 1 2)	58.264	0.5521	6.9374	
119.943	0.8897	1.0	(0 0 6)	59.971	0.5620	7.0621	
121.790	0.8816	1.0	(3 0 3)	60.895	0.5672	7.1270	
130.881	0.8469	1.0	(3 1 3)	65.440	0.5904	7.4190	
131.568	0.8446	2.0	(3 2 0)	65.784	0.5920	7.4392	
132.889	0.8403	4.0	(2 1 5)	66.444	0.5950	7.4773	
133.584	0.8381	5.0	(2 2 4)	66.792	0.5966	7.4969	
134.816	0.8343	4.0	(3 2 1)	67.408	0.5993	7.5311	
138.984	0.8224	2.0	(1 1 6)	69.492	0.6080	7.6401	

PDF#46-1043: QM=Uncommon(?); d=Diffractometer; I=(Unknown)

materials MDPI

Palladium, syn
Pd
Radiation=CuKa1 Lambda=1.5406 Filter=
Calibration= 2T=40.118-124.627I/Ic(RIR)=4.1
Ref: Level-1 PDF

Cubic, Fm3m(225) Z=4 mp=
CELL: 3.89019 x 3.89019 x 3.89019 <90.0 x 90.0 x 90.0> P.S=
Density(c)=12.004 Density(m)= Mwt= Vol=58.9
Ref: Ibid.

Strong Lines: 2.25/X 1.95/6 1.17/6 1.38/4 0.89/4 1.12/2 0.97/1 0.87/1

2-Theta	d(?)	I(f)	(h k l)	Theta	1/(2d)	2pi/d	n^2
40.118	2.2458	100.0	(1 1 1)	20.059	0.2226	2.7977	3
46.658	1.9451	60.0	(2 0 0)	23.329	0.2571	3.2302	4
68.119	1.3754	42.0	(2 2 0)	34.059	0.3635	4.5684	8
82.098	1.1730	55.0	(3 1 1)	41.049	0.4263	5.3567	11
86.617	1.1230	15.0	(2 2 2)	43.308	0.4452	5.5951	12
104.751	0.9725	13.0	(4 0 0)	52.376	0.5141	6.4606	16
119.329	0.8925	40.0	(3 3 1)	59.665	0.5602	7.0402	19
124.627	0.8699	11.0	(4 2 0)	62.313	0.5748	7.2230	20
